# Components of School-Based Interventions Stimulating Students’ Intrapersonal and Interpersonal Domains: A Meta-analysis

**DOI:** 10.1007/s10567-020-00328-y

**Published:** 2020-10-03

**Authors:** Esther Mertens, Maja Deković, Patty Leijten, Monique Van Londen, Ellen Reitz

**Affiliations:** 1 Utrecht University, Child and Adolescent Studies, Utrecht, The Netherlands; 2 University of Amsterdam, Child Development and Education, Amsterdam, The Netherlands

**Keywords:** Components, School-based intervention, Intrapersonal domain, Interpersonal domain, Students

## Abstract

Many universal school-based interventions aim to stimulate students’ intrapersonal (e.g., self-esteem) and interpersonal (e.g., school climate) domains. To improve our understanding of why some of these interventions yield stronger effects than others, we identified intervention components that are related to stronger or weaker intervention effects. We systematically searched four databases (i.e., PsycINFO, PubMed, ERIC, CENTRAL) for controlled evaluations of universal school-based interventions. In total, 104 included studies (529 included effect sizes) reported on 99 unique interventions. Interventions showed small positive effects on the intrapersonal (*d* = 0.19) and interpersonal (*d* = 0.15) domains. Focusing on self-awareness and problem solving, using more active learning approaches, and using more extensive interventions predicted stronger intervention effects on aspects of both domains. In contrast, efforts to improve emotion regulation, assertiveness, cognitive coping, and using group discussions predicted weaker intervention effects. Furthermore, commonly implemented components were not necessarily related to stronger intervention effects and components that were related to stronger effects were not necessarily often implemented. Our findings highlight the need to carefully select components for inclusion in interventions.

PROSPERO Registration Number: CRD42019137981.

## Introduction

Schools are expected to foster not only their students’ cognitive development, but also their students’ wellbeing. Schools should implement policies and practices striving to improve students’ attitudes, values, and social support (Langford et al. 2014; World Health Organization 1995). To this end, a range of universal school-based interventions have been developed to enhance students’ intrapersonal and interpersonal domains. The intrapersonal domain refers to managing one’s own feelings, emotions, and attitudes pertained to the individual self (Barber 2005). The interpersonal domain refers to the ability to build and maintain positive relationships with others and to understand social situations, roles, and norms, and respond appropriately (Pellegrino and Hilton 2012; Shek and Leung 2016). Both domains are intertwined as the way individuals view themselves can influence how they approach social interactions and vice versa (Finkel and Vohs 2006).

Even though the two domains are related, they are also meaningfully distinct. While the intrapersonal domain represents an individual’s subjective psychological functioning, the interpersonal domain represents an individual’s social functioning (Dufner et al. 2019). This distinction is empirically supported by factor and profile analyses (e.g., Gilman and Anderman 2006; Park et al. 2017), and by relating the two domains to various developmental outcomes. For instance, competencies in the intrapersonal domain predict better academic achievement and competencies in the interpersonal domain predict better peer relations (Park et al. 2017). While students can acquire competencies in both domains by mastering relevant cognitive, affective, and social skills (Durlak et al. 2011), difficulties with these skills can set students at increased risk of developing problems in the intrapersonal domain, such as internalizing behavior, or in the interpersonal domain, such as aggression (Modecki et al. 2017; White et al. 2013). Given that competencies in both domains are markers of a healthy psychosocial development, whereas problems in these domains increase the likelihood of developing psychopathology later in life, it is important to stimulate youth’s development in these domains (Van Order et al. 2005).

Children’s intra- and interpersonal domains develop throughout their youth, but the importance of these skills becomes particularly pronounced in adolescence when they consolidate their own identity and peer relationships become increasingly important. Adolescents spend less time at home and longer hours at school which provides them increasing opportunities and requirements to interact with others, such as peers, teachers, and romantic partners (Barber 2005). This makes secondary school a potentially good target for interventions to foster youth’s intra- and interpersonal domains. In the present meta-analysis, we therefore examined the effects of universal secondary school-based interventions on students’ intrapersonal and interpersonal domains.

School-based interventions addressing adolescents’ intra- and interpersonal domains typically show small positive effects (e.g., effect sizes (Cohen’s *d*) ranging from 0.03 to 0.24; Dray et al. 2017; Durlak et al. 2011; Jiménez-Barbero et al. 2016). One way to increase our understanding of when interventions are most effective is by studying which components are related to intervention effects. If we can identify components associated with stronger (or weaker) intervention effects, this could help generate hypotheses about the components that drive intervention effects, and thus about how interventions could be improved. In addition, schools can make informed decisions about which intervention to implement, by selecting interventions based on the evidence base for the components. As a first step towards generating hypotheses about components that drive intervention effects, Boustani et al. (2015) listed components that are most frequently included in effective school-based interventions (e.g., problem solving, psychoeducation). Although such a frequency count provides a useful overview, it does not show whether the effectiveness of interventions relates to the presence of the components. Furthermore, due to the focus on effective interventions, the overview cannot identify components related to weaker intervention effects. The present meta-analysis statistically tested which components are related to stronger or weaker intervention effects.

In the literature, typically three types of components are distinguished: Content, instructional, and structural components. *Content components* are specific skills adolescents learn to promote positive outcomes, such as emotion regulation and problem solving (Boustani et al. 2015), i.e., “what they learn.” *Instructional components* are techniques and methods of information delivery used by the intervention facilitator, such as cognitive restructuring and modeling (Boustani et al. 2015), i.e., “how they learn it.” *Structural components* describe the structure of the intervention that might impact results, such as the number of sessions and whether or not parents are included in the intervention (Lee et al. 2014), i.e., “how the intervention is set up.” By examining all three types of components, we strive to improve our understanding of whether a certain type of component is particularly associated with intervention effects.

Various meta-analyses have successfully identified intervention components that predict intervention effects (e.g., De Mooij et al. 2020; De Vries et al. 2015; Kaminski et al. 2008; Van der Put et al. 2018), but few meta-analyses have focused on components of school-based interventions. Prior meta-analyses that did examine components of school-based interventions focused on substance use, sexual risk behaviors (e.g., pregnancy, STD/HIV) and/or nutrition (see for a review of reviews Peters et al. 2009). For example, Onrust et al. (2016), focusing on substance use, found that components that sought to stimulate students’ self-control and problem solving, and components that included cognitive restructuring, adjusting social norms (e.g., peer education), and parental involvement predicted stronger substance use reductions. Hennessy and Tanner-Smith (2015), focusing on alcohol use, found that components that included an individual and motivational enhancement approach predicted stronger alcohol use reduction.

In the present meta-analysis, we examined which components are related to stronger (or weaker) school-based intervention effects on students’ intra- and interpersonal domains. We focused on a broad range of outcomes for two reasons. First, many school-based interventions aim to enhance multiple aspects of students’ development (e.g., promoting self-efficacy, psychological wellbeing, and life satisfaction: Gigantesco et al. 2015; bullying, aggression, and wellbeing across various domains: Bonell et al. 2018). We wanted to align with this approach in our meta-analysis. Second, some components may be related to intervention effects on some outcomes, but not on others. Unraveling these differential associations may provide insight in the extent to which interventions need to be matched to specific problems.

We studied relations between components and intervention effects across different populations (e.g., socio-economically advantaged students, predominately ethnic minority students). Although the effectiveness of components may depend on participant characteristics, any moderator effects by participant characteristics were beyond the scope of this study—our goal was to provide a first overview of which components are related to intervention effects. We analyzed all three types of components (i.e., content, instructional, and structural) and tested whether interventions with a specific component showed larger (or smaller) effect sizes than interventions without that component, using multilevel meta-regression. This enabled us to identify not only which components were associated with stronger effects, suggesting potential effective components, but also components associated with weaker effects, suggesting potential ineffective components. Knowing what does *not* work is equally important as knowing what does work (e.g., Poulin et al. 2001; Werch and Owen 2002).

Concerning content components, based on the results of Onrust et al. (2016) and Boustani et al. (2015), we hypothesized that basic life skills and self-awareness would be related to stronger intervention effects on students’ intra- and interpersonal domains. Basic life skills refers to abilities for adaptive and positive behavior to deal with demands and challenges of everyday life (World Health Organization 1997). Several reviews suggest the importance of basic life skills, such as problem solving, assertiveness, and social skills, for a range of outcomes of effective school-based interventions (e.g., intra- and interpersonal domains, Boustani et al. 2015; drug use, Cuijpers 2002). Self-awareness indicates a realistic and accurate assessment of one’s strengths and norms, and is related to improvements on the interpersonal domain (e.g., Shek and Leung 2016). Raising self-awareness, such as insight building and self-efficacy, is often used in effective interventions targeting the intrapersonal and interpersonal domains (Boustani et al. 2015).

For instructional components, we hypothesized that components using a more active learning approach, in which students interact with each other and perform tasks (e.g., practicing through role-play), would be related to stronger intervention effects. Active learning approaches have consistently been related to stronger effects. For instance, Kaminski et al. (2008) found in their meta-analysis that parenting interventions in which parents practiced the learned skills were more effective than interventions that did not include practice. Similarly, Cuijpers (2002) concluded in his review of school-based drug interventions that interventions using more active methods (e.g., discussion) were more effective than interventions using more passive methods (e.g., didactic instruction).

Regarding structural components, the general assumption is that longer and extensive interventions are more effective than briefer and less extensive interventions (Yeager and Walton 2011). The evidence, however, is conflicted. Some meta-analyses showed that longer and extensive interventions are indeed more effective. For instance, interventions showed stronger effects as the time span, number of sessions, and involved persons (i.e., whole school, parents) increased (De Vries et al. 2015; Ttofi and Farrington 2011). Other meta-analyses, on the other hand, showed that briefer and less extensive interventions are more effective (i.e., “less is more”). For instance, interventions showed stronger effects when the time span was short, the number of sessions limited, and no additional services were provided (Cuijpers 2002; Kaminski et al. 2008; Van der Put et al. 2018). Longer and extensive interventions require more time and effort to implement with fidelity (Bakermans-Kranenburg et al. 2003); resources that may lack in many schools. Given that findings of previous research concerning structural components are inconclusive, examining the relations between these components and intervention effects was explorative.

In summary, identifying components related to stronger or weaker intervention effects has both theoretical and practical implications. First, it expands our knowledge concerning interventions. We begin to unravel, based on associations between components and intervention effects, what is more important to change students’ intra- and interpersonal domains: What they learn, how they learn it, or how the intervention is set up? Second, it helps schools to make informed decisions about which intervention to implement and catalyzes hypotheses generation about how interventions may be optimized. We hope this knowledge can be used as a first step towards improving the effectiveness of school-based interventions addressing students’ intra- and interpersonal domains.

## Method

### Inclusion and Exclusion Criteria

We sought to include evaluations of universal secondary school-based interventions addressing students’ intrapersonal and interpersonal domains. Universal secondary school-based interventions were defined as interventions delivered to students during regular school hours, targeting all students (Mychailyszyn et al. 2012; Peters et al. 2009). The intrapersonal domain was defined as managing one’s own feelings, emotions, and attitudes pertained to the individual self (Barber 2005) in which one can experience competencies (e.g., resilience, self-esteem, self-regulation, general wellbeing) and problems (e.g., internalizing behavior). The interpersonal domain was defined as the ability of an individual to build and maintain positive relationships with others and understanding social situations, roles and norms, and respond appropriately (Pellegrino and Hilton 2012; Shek and Leung 2016) in which one can experience competencies (e.g., sexual health, social competence, positive school climate) and problems (e.g., aggression, bullying). Although one’s development in these two domains has been associated with psychopathology, the domains and psychopathology are not opposite ends of the same continuum (e.g., Girard et al. 2017). In the current meta-analysis, we focused on interventions stimulating the development in the intra- and interpersonal domains rather than preventing psychopathology.

Studies were eligible for review when (1) the intervention was implemented in a regular school (i.e., not in special education), (2) the intervention was implemented during regular school hours in a group setting, (3) the intervention was aimed at improving (subdomains of) the intra- and/or interpersonal domain (i.e., interventions primarily aiming to improve students’ physical health (e.g., prevention of substance use, nutrition, pregnancy, STDs) or prevent psychopathology (e.g., depression) were excluded.), (4) the intervention was universal, so targeting all students, (5) the participants were in middle school or high school (Grades 6–12), (6) the study included a control group, (7) the study included a quantitative baseline and post intervention measurement of (subdomains of) the intrapersonal domain and/or interpersonal domain, (8) sufficient information concerning baseline and post intervention measurements was reported, or obtained after contact with the author, so that effect sizes could be calculated post intervention, corrected for baseline differences, (9) the study was written in English, and (10) the study was published as article, book, or book chapter. Research has shown that including unpublished studies does not reduce the possible impact of publication bias and is sometimes even counterproductive due to selection bias (Ferguson and Brannick 2012).

### Literature Search

We searched four databases (i.e., PsycINFO, PubMed, ERIC, and CENTRAL). With these databases we searched the psychological, medical, and educational literature, and (quasi-)randomized controlled trials specifically. The search was not restricted to a time period. Search terms were used to elicit school-based interventions (e.g., school, class), interventions (e.g., prevention, intervention), adolescents (e.g., adolescent, youth), and intra- and interpersonal outcomes (e.g., self-esteem, social competence). Because these search terms led to an extremely high number of studies, we added some restrictions to the search, to avoid picking up interventions targeting other populations (e.g., preschool, clinical) or domains (e.g., substance use, lifestyle) than targeted in this study. The complete list of search terms is provided in “Appendix A”. This search (April 2019) resulted in 6102 studies in PsycINFO, 2964 studies in PubMed, 1683 studies in ERIC, and 567 studies in CENTRAL. Removal of duplicates resulted in 9,498 unique studies. In addition, reference lists of included studies and identified relevant reviews and meta-analyses were searched. This resulted in 22 additional studies.

**Fig. 1:**
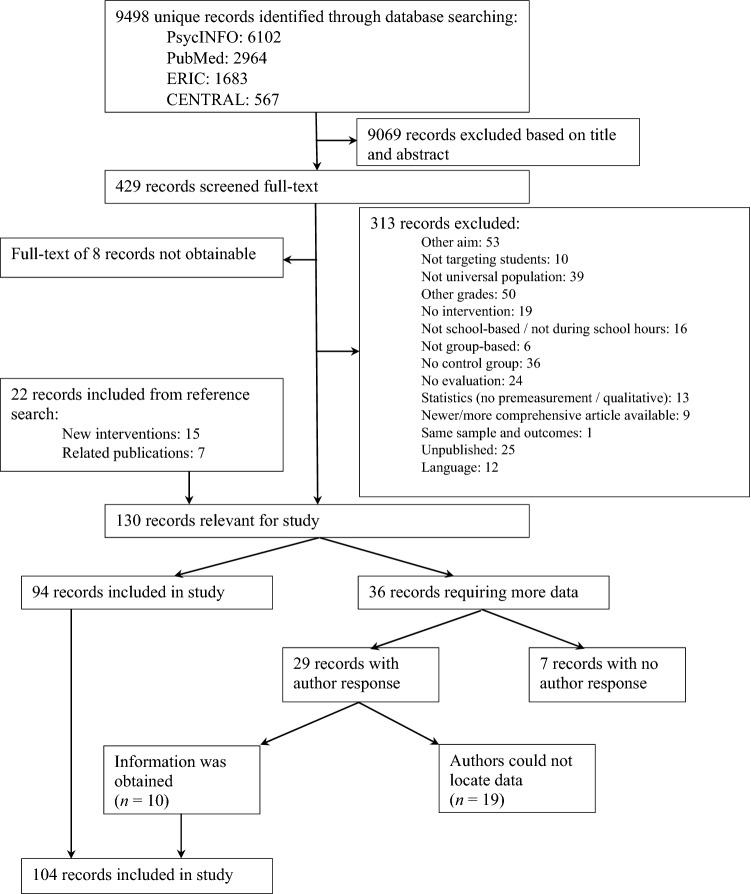
Flow diagram

All studies identified by the search were first screened for eligibility based on their title and abstract. Title and abstract were reviewed to assess whether the study met inclusion and/or exclusion criteria. We specifically focused on information where the intervention was conducted (e.g., school-based), at what type of school the intervention was conducted (e.g., middle/high school), and whether the intervention was implemented during regular school hours (rather than after school). Based on this screening 9,068 studies (95%) were excluded. The remaining 429 studies were studied full-text. In this second screening phase another 310 studies (72%) were excluded. See Fig. 1 for the flow diagram.

In order to assess reliability of these two screening phases, a research assistant independently screened a random selection of 9% (800 studies) of all identified studies concerning the first screening phase and in the second screening phase 10% (45 studies) of the remaining studies. The research assistant judged the relevance of the studies for the current meta-analysis based on the inclusion and exclusion criteria. Reliability was substantial, with 98% agreement (Cohen’s κ = 0.71; Landis and Koch 1977) regarding the first screening phase and 89% agreement (Cohen’s κ = 0.76; Landis and Koch 1977) regarding the second screening phase. Any disagreements between the researchers concerning inclusion were solved through discussion.

### Data Extraction

Studies were coded for information concerning the study (e.g., year of publication, country where study was conducted), sample (e.g., age, gender distribution), design and method (e.g., randomization, attrition analyses), intervention (e.g., intervention provider, aim of intervention), effect size data (e.g., outcome category), and intervention components (e.g., problem solving, practice, parental involvement). The intervention components were primarily based on the meta-analysis by Boustani et al. (2015) who, in turn, based their components on the PracticeWise Clinical Coding System (PracticeWise 2009). Additionally, we reviewed the components of the reviews and meta-analyses of Kaminski et al. (2008), Onrust et al. (2016), Peters et al. (2009), and Van der Put et al. (2018) to further strengthen the theoretical base of the components. Based on this broad theoretical basis, we adjusted some components of Boustani et al. (2015) due to highly overlapping content and co-occurrence (i.e., we combined *communication skills* and *social skills*; we combined *cognitive coping* and *coping skills*; *emotion regulation* contains *anger management*; *practice* contains *role-play*) and we deleted some components due to low frequency (i.e., civic responsibility, support networking). An overview of all components and their definitions is presented in “Appendix B”. Sources cited in the study and other freely available materials, such as descriptions from the developer or websites, were retrieved for coding the components (Boustani et al. 2015; Kaminski et al. 2008). In cases where insufficient data were reported for calculating the effect size, the first author was contacted. When this author had not responded after a reminder, the second or last author was contacted and, if necessary, reminded. If the required data could not be obtained after this, the study was excluded from the meta-analysis (see Fig. 1 for the flow diagram).

Of the included studies, 28% (30 studies) was coded independently by a second coder for reliability. The inter-rater reliability was moderate to excellent (Landis and Koch 1977) with an average intra-class-correlation of 0.97 (SD = 0.05), ranging from 0.88 to 1.00, for continuous variables, and an average Cohen’s kappa of 0.82 (SD = 0.11), ranging from 0.60 to 1.00, for categorical variables (see “Appendix C” for the reliability per individual code). Coding of the component ‘Insight building’ was not reliable with Cohen’s kappa of 0.52. Disagreements between the two coders were discussed and solved unanimously.

### Calculation and Analyses of Effect Sizes

Effect sizes were represented as Cohen’s *d*, reflecting the standardized mean difference between the intervention and control condition, following the procedures of Lipsey and Wilson (2001):

$${\text{Cohen's }}d = \frac{{\bar{X}_{{G1}} - \bar{X}_{{G2}} }}{{s_{p} }}$$

Effect sizes were calculated at post intervention (i.e., within 6 months after the intervention) and corrected for baseline differences. Positive effect sizes indicated better results for the intervention compared to the control condition. All effect sizes were adjusted using the Hedges’ (1983) small sample correction prior to analyses:

$$\text{Hedges' adjusted effect size }= d *\left(1- \frac{3}{4N-9}\right)$$

Outliers were examined and, when believed to be unrepresentative, winsorized by replacing outliers with the value of the lower or upper value of two standard deviants from the mean (Lipsey and Wilson 2001).

### Publication Bias

As commonly known, studies with nonsignificant or negative results are less likely to be published than studies with significant or positive results. The risk of publication bias was tested using a funnel plot. A funnel plot is a scatter plot in which the effect sizes are plotted against their precision (i.e., standard error). It is assumed that the effect sizes of the studies are symmetrically distributed around the true effect size, with more precise effect sizes (typically those from larger studies) at the top of the funnel and less precise effect sizes (typically those from smaller studies) at the base of the funnel. Asymmetry in the funnel plot can be an indication of publication bias (Light and Pillemer 1984). Whether or not a funnel plot is asymmetrical can be statistically tested with Egger’s regression test (Egger et al. 1997). When the funnel plot is asymmetrical according to Egger’s regression test the trim-and-fill analysis (Duval and Tweedie 2000a, 2000b) can be used to adjust the effect for possible publication bias. This analysis estimates how many studies fall outside the symmetric part of the funnel plot and trims this outlying part. With the remaining symmetric funnel plot the true center of the funnel is estimated. The trimmed studies and their missing counterparts are replaced in the funnel representing imputed ‘missing’ effect sizes. Based on this filled funnel plot, the corrected mean is estimated resulting in an adjusted effect size. Tests to visualize and examine publication bias assume independence of effect sizes, which is not the case in multilevel meta-analyses. We took this violation into account by using the variance of the effect sizes as a moderator in Egger’s regression test.

### Analyses

We calculated an effect size for each reported measure of the intra- or interpersonal domain. To account for the clustering of effect sizes within a trial, we used multilevel meta-analytical models with three levels: Sampling variance around each effect size (level 1), variance between effect sizes within studies (level 2), and variance between studies (level 3; Assink and Wibbelink 2016; Van den Noortgate et al. 2013).

The unit of analyses were the interventions rather than the publications, since we are interested in the effectiveness of the intervention compared to the control condition. When one publication reported on two interventions, both interventions were included and analyzed separately. When multiple publications reported on the same intervention, evaluated in different studies with different samples, their effect sizes were analyzed together, clustered within the same intervention. When multiple publications reported on the same intervention, evaluated in the same study with the same sample, we coded the most comprehensive publication; the less comprehensive publication was checked for additional information and their effect sizes were analyzed together, clustered within the same intervention.

The multilevel analyses were conducted in R using the metaphor package (Viechtbauer 2010). First, the overall effects of universal school-based interventions on students’ intrapersonal and interpersonal domains were estimated in separate models. Methodological rigor was assessed to examine how well the overall effect sizes reflected the effects of the intervention rather than methodological influences or biases (Lipsey and Wilson 2001). Based on the Cochrane Risk of Bias 2.0 tool for Cluster Randomized Trials (Higgins et al. 2016) randomization (random vs. quasi-random assignment) and completeness of outcome data (percentage of drop-out) were analyzed as covariates. Additionally, the type of comparison group (passive: No intervention/waitlist vs. active: Care as usual/other intervention) was examined as covariate to examine absolute versus relative effects of the interventions. Characteristics of methodological rigor that predicted the overall effect sizes were included as covariates in further analyses.

To analyze which components were associated with stronger or weaker intervention effects, moderation analyses were conducted. Moderation analyses were conducted only if both levels of the moderator (i.e., component present or not) contained at least three effect sizes (Crocetti 2016). Note that these moderation analyses are based on correlations between interventions and effects. Besides significant effects (*p* < 0.05), effects with a trend towards significance (*p* < 0.10) were reported. These trends contribute to the hypotheses generation nature of the meta-analysis and provide an indication to what extent moderation by a certain component for a certain outcome can be generalized to other outcomes.

## Results

### Descriptive Characteristics

The present meta-analysis included 104 publications reporting on 99 unique interventions. In total, 529 effect sizes were extracted from the publications comparing the intervention with the control condition on the intrapersonal domain (*k* = 218) or the interpersonal domain (*k* = 311). Four effect sizes were extreme outliers, more than four standard deviations above the mean. All were derived from the same study (Haynes and Avery 1979) and believed to be unrepresentatively high. These four effect sizes were therefore winsorized.

The studies, published between 1979 and 2019 (Median publication year: 2013), were conducted in the USA (*k* = 36), Canada (*k* = 2), Europe (*k* = 45), Australia (*k* = 7), Asia (*k* = 13), and Africa (*k* = 1). Most studies randomly assigned participants to the conditions (*k* = 70). In 47 studies, the intervention group was compared to an active control group (i.e., Care As Usual or another intervention). The other 57 studies compared the intervention group to a passive control group (i.e., waitlist or no intervention). In total, the studies comprised 97,884 participants with an average age of 13.70 years (SD = 1.50) at the start of the intervention and mean sex distribution of 49% boys (SD = 16.43). Of the studies reporting ethnicity (59%), participants represented mostly ethnic majority in 59% of the studies, mostly ethnic minorities in 28% of the studies, and mixed ethnic majority and minorities in 13% of the studies. The mean drop-out rate of participants was 12.33% (SD = 10.65). The interventions consisted on average of 14 sessions (SD = 15.35) with a time span of 22.55 weeks (SD = 33.59). Roughly half of the interventions were provided by teachers (*k* = 46) and the other half (*k* = 58) by professionals. In addition, in 6 interventions the intervention was (also) provided by a peer. “Appendix D” provides the key characteristics of the included studies.

### Overall Effect Sizes

Interventions had a small positive effect on students’ intrapersonal domain [*d* = 0.19, 95% CI (0.13; 0.25)]. More specifically, the positive intervention effects for self-esteem and self-regulation were somewhat stronger than for internalizing problems and wellbeing. No significant intervention effect was found for resilience (see Table 1). Interventions also had a small positive effect on students’ interpersonal domain [*d* = 0.15, 95% CI (0.10; 0.19)]. The magnitude of intervention effects was fairly similar for aggression, sexual health, social competence, and bullying. Interventions showed the strongest positive effects on school climate. However, this effect did not reach significance due to the small number of effect sizes for this subdomain. Definitions of the two general domains and the subdomains are provided in “Appendix E”.

**Table 1:** Effectiveness of interventions targeting the intra- and interpersonal domains

Domains	Effect sizes (*k*)	Effect size	95% CI
Intrapersonal	218	0.19	0.13; 0.25
Resilience	13	0.06	− 0.01; 0.14
Self-esteem	53	0.25	0.11; 0.39
Self-regulation	33	0.21	0.08; 0.33
General wellbeing	63	0.13	0.08; 0.19
Internalizing problems	50	0.19	0.10; 0.29
Interpersonal	311	0.15	0.10; 0.19
Sexual health	61	0.16	0.07; 0.26
Social competence	63	0.16	0.10; 0.23
School climate	17	0.24	− 0.11; 0.58
Aggression	84	0.10	0.03; 0.17
Bullying	82	0.13	0.03; 0.24

### Publication Bias

**Fig. 2:**
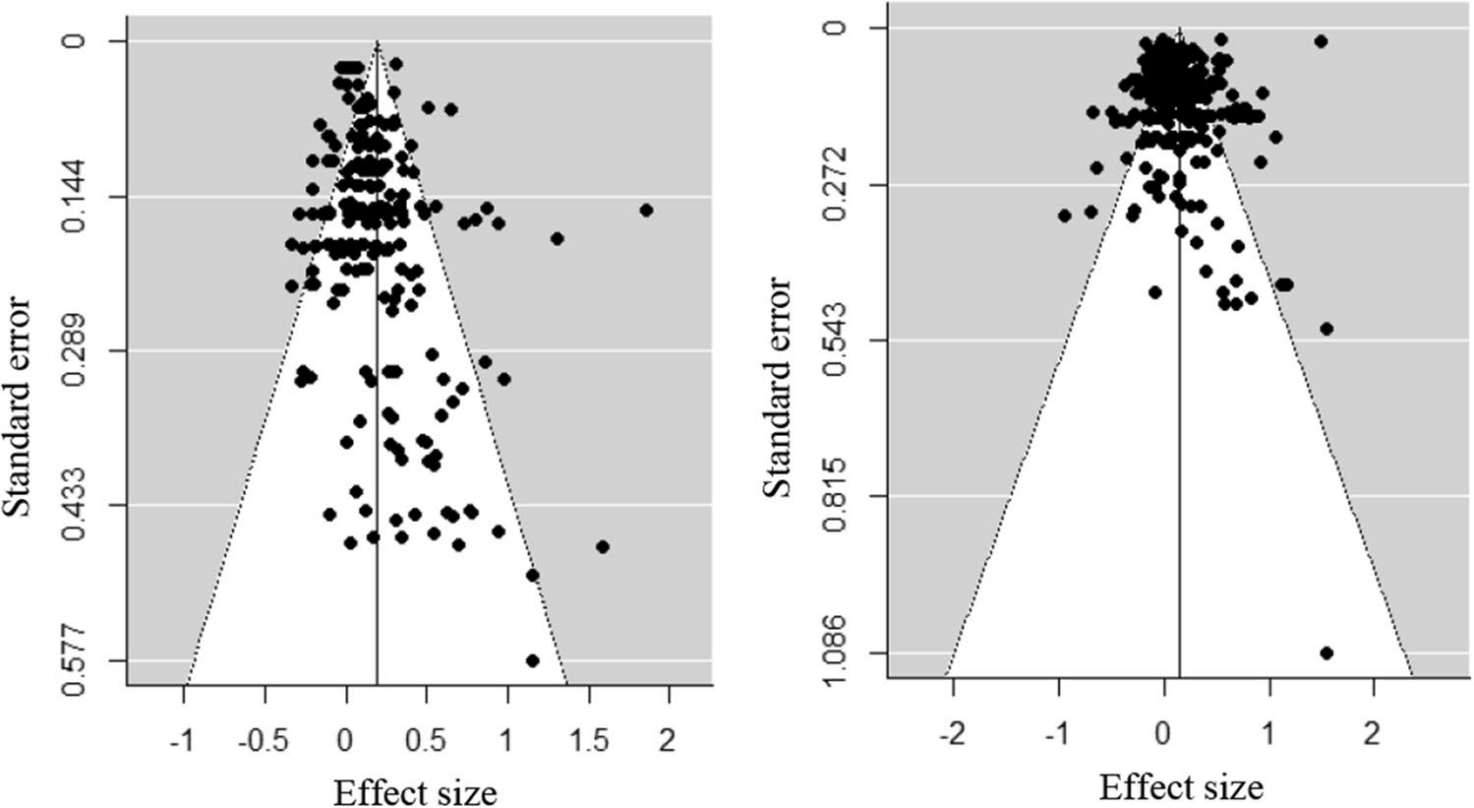
Funnel plot of effect sizes concerning the intrapersonal (left) and interpersonal (right) domains

For both the intra- and interpersonal domains, the distribution of effect sizes appeared to be symmetrical (Egger’s regression test: Intrapersonal *z* =  − 0.22, *p* = 0.826; Interpersonal *z* = 0.17, *p* = 0.862; see Fig. 2), indicating that there was low risk of publication bias.

### Intervention Components Related to Intervention Effects

#### Preliminary Analyses

**Fig. 3:**
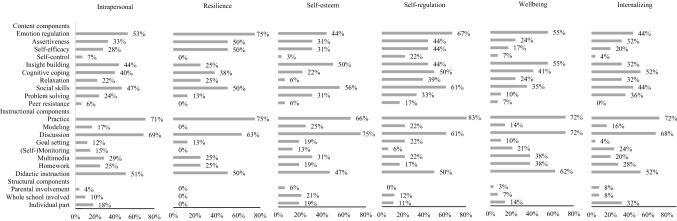
Frequencies of components applied in interventions targeting the intrapersonal domain and subdomains

**Fig. 4:**
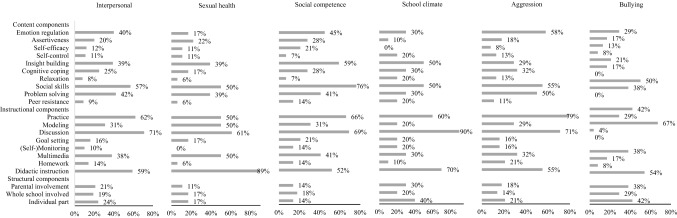
Frequencies of components applied in interventions targeting the interpersonal domain and subdomains

Interventions targeting students’ intrapersonal domain (see Fig. 3) and those targeting students’ interpersonal domain (see Fig. 4) shared many commonly used components. Most commonly used content components are teaching students social skills, emotion regulation, and insight building. Most commonly used instructional components are implementing discussions, practice, and didactic instruction. The most commonly used structural component is additional individual guidance during the intervention.

Concerning methodological rigor, whether or not participants were randomized, drop-out rate, and type of comparison group were not related to effect sizes concerning the intrapersonal domain or the subdomains. Whether or not participants were randomized was related to effect sizes concerning the interpersonal domain; randomized studies yielded stronger effects. Percentage of drop-out was related to effect sizes concerning social competence; studies with lower drop-out rates yielded stronger effects. Whether or not participants were randomized and drop-out rates were also related to effect sizes concerning bullying; randomized studies and higher drop-out rates yielded stronger effects. Therefore, randomization and drop-out were added as covariates when it were significant predictors of the effect size in the moderation analyses concerning the interpersonal domain, social competence, and bullying.

#### Intrapersonal Domain

Of the ten content components, none were significantly related to intervention effects on students’ intrapersonal domain in general (see Table 2). However, there was a trend that teaching emotion regulation was associated with weaker intervention effects on the intrapersonal domain overall, and specifically on self-esteem. For the subdomains, teaching assertiveness was associated with weaker effects on internalizing problems. Furthermore, there was a trend that insight building was associated with stronger effects on resilience.

**Table 2:** Effect sizes of interventions with and without components targeting the intrapersonal domain

Component	Intrapersonal			Resilience			Self-esteem			Self-regulation			Wellbeing			Internalizing		
	#ES	ES	*B*	#ES	ES	*B*	#ES	ES	*B*	#ES	ES	*B*	#ES	ES	*B*	#ES	ES	*B*
Content components																		
Emotion regulation																		
Yes	116	0.14	− 0.11^†^	11	–	–	26	0.13	− 0.24^†^	18	0.20	− 0.03	27	0.12	− 0.05	23	0.17	− 0.03
No	102	0.25		2	–		27	0.37		15	0.23		36	0.16		27	0.21	
Assertiveness																		
Yes	61	0.18	− 0.02	9	0.12	0.08	14	0.25	− 0.00	9	0.07	− 0.22	12	0.15	0.02	15	0.04	− 0.21^*^
No	157	0.20		4	0.04		39	0.26		24	0.29		51	0.13		35	0.26	
Self-efficacy																		
Yes	61	0.20	0.00	9	0.07	− 0.02	19	0.23	− 0.04	10	0.13	− 0.14	12	0.14	0.02	10	0.08	− 0.15
No	157	0.19		4	0.09		34	0.27		23	0.27		51	0.13		40	0.23	
Self-control																		
Yes	18	0.07	− 0.13	0	–	–	2	–	–	9	0.15	− 0.08	5	0.05	− 0.09	2	–	–
No	200	0.20		13	–		51	–		24	0.23		58	0.14		48	–	
Insight building																		
Yes	92	0.18	− 0.03	7	0.22	0.18^†^	24	0.20	− 0.11	15	0.29	0.15	30	0.12	− 0.03	16	0.28	0.13
No	126	0.21		6	0.04		29	0.31		18	0.14		33	0.15		34	0.15	
Cognitive coping																		
Yes	90	0.15	− 0.07	3	0.04	− 0.11	12	0.22	− 0.05	22	0.13	− 0.18	20	0.15	0.03	28	0.15	− 0.09
No	128	0.22		10	0.15		41	0.26		11	0.31		43	0.12		22	0.23	
Relaxation																		
Yes	50	0.17	− 0.03	2	–	–	4	0.06	− 0.21	12	0.19	− 0.03	18	0.10	− 0.04	14	0.19	− 0.01
No	168	0.20		11	–		49	0.27		21	0.22		45	0.14		36	0.20	
Social skills																		
Yes	99	0.20	0.02	9	0.07	− 0.02	31	0.31	0.13	17	0.23	0.07	21	0.09	− 0.07	21	0.16	− 0.07
No	119	0.18		4	0.09		22	0.18		16	0.16		42	0.17		29	0.23	
Problem solving																		
Yes	51	0.24	0.07	6	0.19	0.15	17	0.34	0.12	7	0.30	0.13	5	0.19	0.06	17	0.20	0.01
No	167	0.17		7	0.04		36	0.21		26	0.17		58	0.13		33	0.19	
Peer resistance																		
Yes	8	0.17	− 0.02	0	–	–	2	–	–	3	0.27	0.08	3	0.13	− 0.01	0	–	–
No	210	0.20		13	–		54	–		30	0.20		60	0.13		50	–	
Instructional components																		
Practice																		
Yes	148	0.24	0.12^*^	11	–	–	34	0.34	0.23	21	0.24	0.11	42	0.14	0.01	39	0.22	0.09
No	70	0.12		2	–		19	0.11		12	0.12		21	0.13		11	0.13	
Modeling																		
Yes	34	0.16	− 0.05	0	–	–	13	0.26	0.00	5	0.08	− 0.15	7	0.14	0.01	9	0.08	− 0.13
No	184	0.20		13	–		40	0.25		28	0.23		56	0.13		41	0.21	
Discussion																		
Yes	143	0.20	0.01	10	0.13	0.09	40	0.21	− 0.20	16	0.26	0.12	42	0.14	0.01	33	0.21	0.05
No	75	0.19		3	–^a^		13	0.41		17	0.14		21	0.13		17	0.16	
Goal setting																		
Yes	27	0.15	− 0.06	6	0.19	0.15	8	0.16	− 0.12	4	0.01	− 0.24	9	0.12	− 0.01	1	–	–
No	191	0.20		7	0.04		45	0.28		29	0.25		54	0.14		49	–	
(Self-)monitoring																		
Yes	29	0.25	0.07	0	–	–	5	0.44	0.21	1	–	–	10	0.10	− 0.04	13	0.35	0.19
No	189	0.18		13	–		48	0.23		32	–		53	0.14		37	0.16	
Multimedia																		
Yes	60	0.16	− 0.06	2	–	–	21	0.16	-0.15	4	0.18	− 0.03	24	0.15	0.04	8	0.19	− 0.00
No	158	0.22		11	–		32	0.31		29	0.21		39	0.12		42	0.19	
Homework																		
Yes	50	0.21	0.03	2	–	–	7	0.34	0.10	4	0.17	− 0.05	21	0.17	0.05	16	0.24	0.07
No	168	0.19		11	–		46	0.24		29	0.21		42	0.12		34	0.18	
Didactic instruction																		
Yes	116	0.19	− 0.01	4	0.09	0.02	28	0.28	0.05	16	0.17	− 0.07	41	0.14	0.02	26	0.23	0.07
No	102	0.20		9	0.07		25	0.23		17	0.25		22	0.12		24	0.16	
Structural components																		
Parental involvement																		
Yes	6	0.18	− 0.01	0	–	–	2	–	–	0	–	–	1	–	–	3	0.29	0.11
No	212	0.19		13	–		51	–		38	–		62	–		47	0.18	
Whole school involving																		
Yes	20	0.22	0.02	1	–	–	10	0.20	− 0.11	2	–	–	6	0.11	− 0.03	3	0.51	0.36^**^
No	198	0.20		12	–		43	0.31		31	–		57	0.13		47	0.15	
Individual part																		
Yes	48	0.19	− 0.00	0	–	–	11	0.23	− 0.03	11	0.15	− 0.07	7	0.12	− 0.01	16	0.22	0.04
No	170	0.19		13	–		42	0.26		22	0.22		56	0.13		34	0.18	
Number of sessions																		
I		0.20	− 0.00		0.11	− 0.01		0.27	− 0.04		0.19	0.05		0.13	− 0.03		0.22	0.20**
Number of components																		
I		0.20	− 0.01		0.09	0.08		0.26	− 0.04		0.22	− 0.03		0.13	− 0.01		0.19	− 0.01

*ES* effect size, *B* meta-regression coefficient, *I* intercept

^a^Did not converge

Differences in effect sizes between interventions with and without that component significant at level of ^†^*p* < 0.10; *p < 0.05; **p < 0.01

Of the eight instructional components, practicing during the intervention was related to significantly stronger intervention effects on students’ intrapersonal domain overall. None of the other components were significantly related to intervention effects on students' intrapersonal domain in general.

Of the five structural components, none were related to intervention effects on students’ intrapersonal domain in general. For the subdomains, associations with stronger effects were found on internalizing problems when the whole school was involved and when the intervention had more sessions.

In sum, interventions that included insight building, where students practiced during the sessions, that involved the whole school staff, and those that had more sessions showed stronger effects for stimulating subdomains of the intrapersonal domain than interventions that did not include these components. Interventions that taught emotion regulation and assertiveness showed weaker effects for stimulating subdomains of the intrapersonal domain than interventions that did not include these components.

#### Interpersonal Domain

Of the ten content components, teaching problem solving was related to stronger intervention effects on students’ interpersonal domain in general, specifically for interventions targeting bullying and school climate (see Table 3). Teaching cognitive coping was related to weaker intervention effects on students’ interpersonal domain overall, and specifically for interventions targeting bullying. Furthermore, there was a trend that insight building was associated with stronger intervention effects on students’ interpersonal domain overall. The other components were not related to intervention effects on the interpersonal domain in general. For the subdomains, insight building was associated with stronger effects on social competence and bullying. Teaching emotion regulation and assertiveness were associated with weaker effects on respectively bullying and aggression.

**Table 3:** Effect sizes of interventions with and without components targeting the interpersonal domain

Component	Interpersonal^a^			Sexual health			Social competence^b^			School climate			Aggression			Bullying^c^		
	#ES	ES	*B*	#ES	ES	*B*	#ES	ES	*B*	#ES	ES	*B*	#ES	ES	*B*	#ES	ES	*B*
Content components																		
Emotion regulation																		
Yes	127	0.04	− 0.06	55	0.00	− .0.19	31	0.17	− 0.03	5	− 0.04	− .0.39	53	0.10	− 0.01	52	0.03	− .0.16^†^
No	184	0.11		6	0.19		32	0.20		12	0.35		31	0.10		30	0.18	
Assertiveness																		
Yes	52	0.09	0.02	9	0.23	0.08	14	0.09	− .0.14	3	0.46	0.26	15	− 0.05	− 0.19*	11	0.23	0.12
No	259	0.08		52	0.15		49	0.23		14	0.21		69	0.13		71	0.11	
Self-efficacy																		
Yes	37	0.11	0.04	5	0.28	0.13	18	0.16	− 0.01	0	–	–	8	− 0.04	− 0.15	5	0.34	0.23
No	274	0.07		56	0.15		45	0.16		17	–		76	0.11		77	0.11	
Self-control																		
Yes	25	0.17	0.10	5	− 0.03	− 0.22	2	–	–	3	0.18	− 0.07	9	0.24	0.16	6	0.38	0.27
No	286	0.07		56	0.19		61	–		14	0.25		75	0.08		76	0.11	
Insight building																		
Yes	118	0.12	0.10^†^	20	0.18	0.03	41	0.24	0.16*	7	0.09	− 0.29	20	0.08	− 0.04	26	0.56	0.55**
No	193	0.03		41	0.15		22	0.08		10	0.38		64	0.11		56	0.02	
Cognitive coping																		
Yes	84	− 0.02	− 0.13*	23	0.07	− 0.11	49	0.12	− 0.10	5	− 0.01	− 0.35	24	0.09	− 0.02	18	0.13	− 0.32*
No	227	0.11		38	0.18		14	0.21		12	0.34		60	0.11		64	0.19	
Relaxation																		
Yes	20	0.08	− 0.00	3	− 0.07	− 0 .24	3	0.06	− 0.15	4	0.18	− 0.07	10	0.29	0.21^†^	0	–	–
No	291	0.08		58	0.18		60	0.21		13	0.25		74	0.08		82		
Social skills																		
Yes	185	0.08	0.01	23	0.10	− .0.12	51	0.19	0.01	9	0.13	− 0.20	49	0.11	0.02	51	0.19	0.12
No	126	0.08		38	0.22		12	0.18		8	0.33		35	0.09		31	0.08	
Problem solving																		
Yes	145	0.21	0.12*	39	0.19	0.04	24	0.16	0.00	6	0.67	0.63^†^	48	0.13	0.06	27	0.30	0.27**
No	166	0.10		22	0.15		39	0.16		11	0.04		36	0.07		55	0.03	
Peer resistance																		
Yes	21	0.08	0.00	3	0.18	0.02	6	0.21	0.06	4	− 0.02	− 0.32	8	− 0.02	− 0.13	0	–	–
No	290	0.08		58	0.16		57	0.15		13	0.30		76	0.12		82	–	
Instructional components																		
Practice																		
Yes	187	0.10	0.04	22	0.14	− 0.06	41	0.19	0.08	11	0.25	0.03	69	0.11	0.02	42	− 0.01	0.13
No	124	0.06		39	0.19		22	0.11		6	0.22		15	0.09		40	− 0.14	
Modeling																		
Yes	95	0.07	− 0.01	22	0.15	− 0.02	13	0.16	− 0.03	3	− 0.11	− 0.42	23	0.15	0.07	34	0.10	− 0.05
No	216	0.08		39	0.18		50	0.20		14	0.31		61	0.08		48	0.15	
Discussion																		
Yes	212	0.08	− 0.02	27	0.14	− 0.07	42	0.21	0.07	14	0.26	0.24	64	0.06	− 0.13^†^	61	− 0.04	0.14
No	99	0.09		34	0.21		21	0.14		3	0.02		20	0.20		21	− 0.18	
Goal setting																		
Yes	45	0.10	0.03	6	0.13	− 0.05	15	0.16	0.00	5	0.02	− 0.27	16	0.04	− 0.07	2	–	–
No	266	0.07		55	0.17		48	0.16		12	0.29		68	0.11		80	–	
(Self-)monitoring																		
Yes	30	0.12	0.04	0	–	–	12	0.13	− 0.04	5	− 0.02	− 0.32	13	0.16	0.07	0	–	–
No	281	0.07		61			51	0.17		12	0.30		71	0.09		82		
Multimedia																		
Yes	115	0.09	0.02	22	0.23	0.14	24	0.28	0.15*	6	0.05	− 0.27	26	0.08	− 0.03	35	0.16	0.05
No	196	0.07		39	0.10		39	0.13		11	0.32		58	0.11		47	0.12	
Homework																		
Yes	30	0.14	0.07	3	0.18	0.02	5	0.33	0.15	2	–	–	15	0.18	0.09	4	0.37	0.26
No	281	0.07		58	0.16		58	0.18		15	–		69	0.09		78	0.11	
Didactic instruction																		
Yes	177	0.12	0.09^†^	58	0.17	0.07	27	0.17	0.02	13	0.36	0.44	44	0.16	0.13^†^	35	0.14	0.01
No	134	0.03		3	0.10		36	0.15		4	− 0.08		40	0.03		47	0.13	
Structural components																		
Parental involvement																		
Yes	62	0.12	0.05	16	0.11	− 0.06	5	0.03	− .0.18	6	0.63	0.57^†^	16	0.10	− 0.01	19	0.20	0.12
No	249	0.07		45	0.17		58	0.20		11	0.06		68	0.10		63	0.09	
Whole school involved																		
Yes	52	0.09	0.02	19	0.06	− 0.13	6	0.03	− 0.18	4	0.98	0.94**	9	0.14	0.03	13	0.35	0.32**
No	259	0.08		42	0.19		57	0.20		13	0.04		75	0.11		69	0.03	
Individual part																		
Yes	56	0.10	0.02	7	0.06	− 0.13	5	0.13	− 0.06	6	0.62	0.63*	14	0.24	0.17^†^	24	0.11	− 0.05
No	255	0.08		54	0.19		58	0.20		11	− 0.02		70	0.07		58	0.15	
Number of sessions																		
I		0.07	0.01		0.11	− 0.14		0.22	0.16		0.16	0.16		0.11	0.04		− 0.09	0.20**
Number of components																		
I		0.07	0.03		0.16	− 0.03		0.19	0.02		0.24	− 0.02		0.10	0.02		0.16	0.10^†^

*ES* effect size‚ *B* meta-regression coefficient, *I* intercept

^a^Analyses corrected for ‘randomization’, except the analysis concerning ‘problem solving.’

^b^Analyses corrected for ‘drop-out’, except the analyses concerning ‘problem solving’, ‘self-efficacy’, ‘didactic instruction’, ‘practice’, ‘goal setting’, and ‘(self-)monitoring.’

^c^Analysis concerning ‘insight building’ corrected for ‘drop-out’, analyses concerning ‘practice’, ‘discussion’, and ‘number of sessions’ corrected for ‘randomization.’

Differences in effect sizes between interventions with and without that component significant at level of ^†^*p* < 0.10; *p < 0.05; **p < 0.01

Of the eight instructional components, none of the components were related to intervention effects on the interpersonal domain in general. Regarding the subdomains, using multimedia was related to stronger effects on social competence. In addition, there were trends that using didactic instruction and relaxation were associated with stronger effects and using discussion had weaker effects on aggression.

Of the five structural components, none were related to intervention effects on students’ interpersonal domain in general. Concerning the subdomains, three components were related to stronger intervention effects. Interventions that included more sessions were associated with stronger effects on bullying. Interventions that involved the whole school were associated with stronger effects on bullying and school climate. Interventions with additional individual guidance were associated with stronger effects on school climate and showed a trend that it was related to stronger effects on aggression. Furthermore, there was a trend that interventions that involved parents were associated with stronger effects on school climate and that interventions that included more components were related to stronger effects on bullying.

In sum, interventions that taught insight building, and problem solving, used didactic instruction, relaxation, and multimedia, involved the whole school and parents, included additional individual guidance, more sessions, and more components showed stronger intervention effects for stimulating subdomains of the interpersonal domain than interventions that did not include these components. Interventions that taught emotion regulation, and assertiveness, and applied cognitive coping, and discussions showed weaker intervention effects for stimulating subdomains of the interpersonal domain than interventions that did not include these components.

## Discussion

It is important to understand the intervention components that contribute to intervention effectiveness, or ineffectiveness, in order to guide intervention selection and implementation. Schools strive to improve their students’ wellbeing, but their time and resources to invest in interventions are limited. This meta-analysis aimed to identify the intervention components that contribute to the effectiveness of universal secondary school-based interventions aiming to stimulate students intra- and interpersonal domains. In line with previous meta-analyses examining universal school-based interventions, we found small positive effects on students’ intra- and interpersonal domains (e.g., Dray et al. 2017; Durlak et al. 2011; Jiménez-Barbero et al. 2016). Overall, none of the discrete components were consistently related to stronger or weaker effects on both students’ intra- and interpersonal domains across the subdomains. In other words, components that were related to stronger or weaker intervention effects typically were so for more specific domains, highlighting the importance of matching intervention to specific competencies or problems. In terms of the type of components that matters most, content components seemed more relevant for stimulating both the intrapersonal domain (e.g., internalizing behavior) and the interpersonal domain (e.g., bullying). Importantly, components related to stronger intervention effects were not necessarily frequently implemented in interventions (e.g., in 10–19% of the interventions). Similarly, components related to weaker intervention effects were generally implemented frequently (e.g., in 40–53% of the interventions).

Content components teaching students self-awareness (i.e., insight building) and problem solving were related to stronger effects, whereas components teaching emotion regulation, assertiveness, and cognitive coping were related to weaker effects. These findings are in line with previous research that indicated teaching self-awareness and problem solving as potential effective components (e.g., Boustani et al. 2015). Teaching emotion regulation, assertiveness, and cognitive coping might be more relevant in different contexts than the secondary school context in which the interventions were implemented. For instance, emotion regulation might be more relevant when implemented in psychotherapy (e.g., Weisz et al. 2012) and teaching assertiveness might be more relevant for students at elementary schools (e.g., Onrust et al. 2016). Teaching cognitive coping was related to weaker effects on interpersonal competences in general. This finding was somewhat surprising, given that cognitive coping is considered an effective component in other interventions as Cognitive Behavioral Therapy (Yovel et al. 2014) with well-trained therapists (Kobak et al. 2017). One possible explanation may be that school-based interventions, are often implemented by teachers who only received a short training (e.g., Challen et al. 2014) and have no to little experience in teaching cognitive coping. Taken together, content components might be differentially related to intervention effects in different contexts.

In general, instructional components that reflect an active learning approach were related to stronger intervention effects (e.g., relaxation, practice). This does not mean that interventions should only use active learning approaches and exclude more passive learning approaches. Interventions that used discussion as method delivery, an active learning component, were related to weaker intervention effects on aggression, whereas interventions using a didactic information delivery as method, a passive learning approach, were related to stronger intervention effects. These findings are in line with the meta-analysis of De Mooij et al. (2020) that showed that psychoeducation was related to stronger effects of Social Skills Training interventions. Using didactic instruction might fit better in the school context than using discussion. In a didactic instruction approach, the emphasis is on knowledge transfer between the teacher and the students, whereas a discussion approach is more dependent on the students and the social skills and cohesiveness of the group. Teachers might be less equipped to prevent a discussion from sidetracking than to teach psychological constructs (Horne et al. 2007).

The results concerning structural components showed that longer and more extensive interventions (e.g., involving parents and the whole school) were more effective for targeting system level outcomes such as school climate. Long-term and extensive interventions might be more effective when the intervention aims to increase students’ feelings of safety at school. By targeting multiple systems in which the students are involved (e.g., school, family) teachers and parents might become more sensitive for problems students encounter, such as bullying or problematic relations with peers (Ttofi and Farrington 2011) and a broad range of risk factors is addressed (Trip et al. 2015). For interventions targeting the individual level such as self-esteem, more extensive interventions were not related to stronger effects nor were less extensive interventions related to weaker effects. Based on these findings, less extensive interventions might be preferred to stimulate the intrapersonal domain due to the easier implementation (Bakermans-Kranenburg et al. 2003), while more extensive interventions may be better suited to stimulate the interpersonal domain.

Furthermore, our results showed that components related to stronger intervention effects were not necessarily commonly implemented. For instance, interventions that involved the whole school were related to stronger effects on internalizing problems, bullying and school climate. However, only 10% to 19% of the included interventions involved the whole school. In contrast, some components that were related to weaker effects are implemented more often. For example, teaching emotion regulation, included in 40% to 53% of the interventions, was related to weaker effect sizes on the intrapersonal domain in general, self-esteem, and bullying. Our frequency counts of components are in line with the frequency count by Boustani et al. (2015) of effective school-based interventions. These findings indicate that it is important to critically consider which components to include in an intervention and to not simply “do what previously has been done.”

Several limitations merit attention. First, we tested associations between components and intervention effectiveness. Based on these associations, we cannot state whether specifically these components are (in)effective or whether other components confounded with that specific component accounted for the association. Moreover, the analyzed components were not implemented in isolation, but in the context of an intervention program consisting of multiple components. Interactions among components can affect their effectiveness. In addition, it remains unclear how the components were implemented, how much time was allotted to certain components, and what the quality of implementation of the component was. These aspects could also influence components’ effectiveness. This meta-analysis should therefore be regarded as hypothesis generating; our results give future research indications which components are interesting to examine further. Future research should test causal individual and synergistic effects of components, and potential order effects of components. Second, the coding of components depended on the sufficiency of the intervention description in the included studies; if a component was not mentioned in the article, or other freely available information concerning the evaluated interventions, it was coded as not present. At the same time, components that are formally part of the intervention, and therefore reported and coded as such, may not necessarily be implemented. It might be that some components were thus coded as “present” while they were not actually implemented. Third, even though we included more than 500 effect sizes, some components (e.g., peer resistance, parental involvement) were less frequently implemented in interventions than other components (e.g., practice, discussion) resulting in better powered analyses for some components than for others. Last, our outcome categories, the two general domains as well as the specific competencies and problems, were relatively broadly defined. On the one hand, these broad outcome categories are a good representation of the broad range of problems that may be present in the heterogeneous student population which schools aim to address with these universal interventions. On the other hand, the outcome categories may be less sensitive to change and could mask associations between specific components and specific outcomes.

In conclusion, when designing and implementing universal school-based interventions, and especially when no rigorous evidence base for the intervention is available, it is important to consider the evidence base of its included components. Some components are often implemented in interventions without being actually related to stronger intervention effects. In fact, some commonly implemented components (e.g., emotion regulation, discussion) were related to weaker intervention effects in our meta-analysis. Vice versa, some components that were related to stronger intervention effects (e.g., involvement of the whole school or parents) were only rarely included in interventions. Thus, it is essential to examine the evidence base of components before including it in an intervention, and to not solely focus on which components have been included in previous interventions. Selecting an intervention for implementation is complex and stakeholders need to take numerous factors into account (e.g., training of providers, match with the context, required resources). We hope our findings can contribute to this process by informing stakeholders which components may be important to be included in an intervention when aiming to address certain competencies or problems; schools can look up which components are associated with stronger effects on outcomes relevant for them and take this evidence-base into consideration in their decision. This meta-analysis provides an empirical foundation for the evidence base of components related to stronger and weaker effects for universal school-based interventions addressing the intra- and interpersonal domain.

## Appendix A—complete list of search terms

**Table:**

Category	Term
Program descriptors	School, schools, class, classroom, classes, school-based, “school based”, group, group-based, “group based”, high-school, “secondary school”
Evaluation descriptors	Intervention, preventive, prevention, program*
Program targets	Adolescent, adolescence, adolescents, youth, teen, teenager, teenagers
Program outcomes	“Psychological well-being”, resilience, resiliency, “emotional adjustment”, “self-efficacy”, “social isolation”, “social identification”, psychosexual, communication, communicative, anger, aggression, aggressive, “social support”, “social safety”, “psychosocial well-being”, “emotion regulation”, “self-regulation”, “self-control”, “self-esteem”, “self-worth”, “self-confidence”, “peer problems”, “relational aggression”, “externalizing behavior”, “externalizing behavior”, “internalizing behavior”, “internalizing behavior”, violence*, “social competence”, “positive social behavior*”, “emotional distress”, “prosocial behavior*”
Restrictions	Substance, drug, drugs, alcohol, smoking, lifestyle, “weight loss”, diet, dietary, obesity, obese, disorder*, disease, clinical, patient, patients, HIV, aids, illness, disability*, disabled, suicide, suicidal, anorexia, eating, pregnancy*, nurse*, neural, neuron, college, university, “primary school”, preschool

## Appendix B—Definitions of Components*

**Table:**

Content components: specific skills adolescents learn to promote positive outcomes	
Emotion regulation	Strategies to help youth identify and appropriately express emotions (including aggression)
Assertiveness	Exercises designed to promote the youth’s ability to assert his or her needs appropriately with others
Self-efficacy	Techniques and training to enhance self-confidence and improve self-efficacy
Self-control	Strategies to help youth interrupt undesired behavioral tendencies (e.g., impulses) and refrain from acting on them
Insight building	Activities specifically designed to help a youth achieve greater self-understanding and adjust attitudes
Cognitive coping	Any techniques designed to alter interpretation of events or deal with stressful situations through examination of the youth’s reported thoughts (e.g., cognitive restructuring)
Relaxation	Techniques or exercises designed to induce physiological calming
Social skills	Training youth how to communicate more effectively with others and providing constructive information, training, and feedback to improve interpersonal verbal or non-verbal functioning
Problem solving	Training in the use of techniques, discussions, or activities designed to bring about solutions to social, emotional, or behavioral problems
Peer resistance	Techniques or training to learn youth how to resist pressure from peers
Instructional components: techniques and methods of information delivery used by the intervention facilitator	
Practice	Practicing of a desired behavior during session (e.g., role-play)
Modeling	Demonstration to the youth of a desired behavior
Discussion	Discussion of topics within a group
Goal setting	The explicit selection of a therapeutic goal for the purpose of working toward achieving that goal
(Self-)monitoring	The repeated measurement of a target index (by the youth)
Multimedia	The use of multimedia to bring or reinforce new knowledge or skills
Homework	Written, verbal, or behavioral assignments to complete between sessions
Didactic instruction	The formal (usually didactic) review of information (e.g., psychoeducation)
Structural components: describe the structure of the intervention that might impact results	
Parental involvement	Parents are directly or indirectly involved during the intervention
Whole school involved	The school staff is directly or indirectly involved during the intervention
Individual part	The intervention includes additional individual guidance or explicit individual progress through the intervention (e.g., expressive writing, internet-based intervention)
Number of sessions	Number of sessions of the intervention
Number of components	Number of components implemented in the intervention

*Definitions are based on Boustani et al. (2015), Kaminski et al. (2008), Onrust et al. (2016), Peters et al. (2009), and Van der Put et al. (2018)

## Appendix C—Reliability Scores of Individual Codes

**Table:**

Descriptive variables	Type of reliability	Reliability
Year of publication	ICC	1.00
Country	Kappa	0.86
Mean age	ICC	1.00
Proportion boys	ICC	0.99
Ethnic composition	Kappa	0.63
Randomization	Kappa	0.80
Drop-out percentage	ICC	1.00
Type of comparison group	Kappa	0.87
Facilitator teacher	Kappa	1.00
Facilitator professional	Kappa	0.85
Facilitator peer	Kappa	1.00
Timespan intervention	ICC	0.99

**Table:**

Components	Type of reliability	Reliability
Content components		
Emotion regulation	Kappa	0.87
Assertiveness	Kappa	0.67
Self-efficacy	Kappa	0.77
Self-control	Kappa	0.87
Insight building	Kappa	0.52
Cognitive coping	Kappa	0.60
Relaxation	Kappa	0.78
Social skills	Kappa	0.80
Problem solving	Kappa	0.87
Peer resistance	Kappa	0.78
Instructional components		
Practice	Kappa	0.66
Modeling	Kappa	0.66
Discussion	Kappa	0.92
Goal setting	Kappa	1.00
Self-monitoring	Kappa	0.87
Multimedia	Kappa	0.92
Homework	Kappa	0.90
Didactic instruction	Kappa	0.62
Structural components		
Parental involvement	Kappa	1.00
Whole school involved	Kappa	0.64
Individual part	Kappa	0.79
Number of sessions	ICC	0.88

## Appendix D—Descriptives of Included Publications

**Table:**

References	Name of intervention	Aim	Target	Category outcomes	Grades
Adler-Beader et al. (2007)	LoveU2: increasing your relationship smarts (RS adapted)	Healthy romantic relationships	Inter	Sexual health, social competence	9–12
Allara et al. (2018)	Diario della salute (my health diary)	Wellbeing and health	Intra	Wellbeing, aggression, school climate	7
Ando et al. (2007)	Adaptation of Going Places Program	Aggressive behavior	Inter	Self-regulation, aggression, social competence, school climate	7
Avery-leaf et al. (1997)	Dating violence prevention program	Dating violence	Inter	Sexual health	11, 12
Baker et al. (2014)	Respect	Sexual violence	Inter	Sexual health	9–12
Barkoukis et al. (2016)	Intervention against Cyberbullying	Cyberbullying	Inter	Social competence, bullying	7–11
Bonell et al. (2018)	Learning Together intervention	Bullying, aggression, and wellbeing	Inter	Wellbeing, aggression, bullying	7
Bosworth et al. (2000)	SMART Talk (based on BARN system)	Problem solving without violence	Inter	Self-esteem, aggression, social competence	6–8
Boulton and Flemington (1996)	Sticks and stones video	Bullying	Inter	Bullying	6–9
Bradley et al. (2010)	TestEdge	Stress, anxiety, wellbeing, and relationships	Intra and inter	Self-regulation, internalizing, wellbeing, social competence, school climate	10
Bull et al. (2009)	The fairplayer.manual	Bullying and relational aggression	Inter	Bullying	9–11
Burckhardt et al. (2016)	Strong Minds	Subjective wellbeing	Intra	Wellbeing	9, 10
Burckhardt et al. (2018)	Dialectical behavior therapy skills group	Mental health symptoms	Intra	Self-regulation, internalizing, aggression	10
Burckhardt et al. (2017)	Acceptance and Commitment Therapy (ACT)	Wellbeing	Intra	Internalizing, wellbeing	10
Calear et al. (2009)	MoodGym	Anxiety and depression	Intra	Internalizing	8–10
Caplan et al. (1992)	The Positive Youth Development Program	Personal and social competence	Intra and inter	Self-esteem, self-regulation, wellbeing, social competence	6, 7
Caprara et al. (2014)	CEPIDEA	Prosocial behavior	Inter	Self-esteem, aggression, social competence	7
Carraro et al. (2014)	Play fighting	Aggressive behavior	Inter	Aggression	8
Castillo-Gualda et al. (2017)	INTEMO (Long version, 3 years)	Aggression	Inter	Wellbeing, aggression	7
Challen et al. (2014)	UK Resilience Program	Resilience	Intra	Internalizing, social competence	6
Chang et al. (2013)	Laughing Qigong Program	Stress	Intra	Self-esteem, wellbeing	7
Cheung and Lee (2010)	Character education	Social competence	Inter	Social competence	8, 9
Coelho et al. (2015)	Positive Attitudes	Social-emotional competence	Intra and inter	Self-esteem, self-regulation, internalizing, social competence	7–9
Coker et al. (2017)	Green Dot violence prevention program	Sexual violence and interpersonal violence	Inter	Aggression	9–12
Connolly et al. (2015)	Respect in Schools Everywhere (RISE)	Bullying, sexual harassment, and date aggression	Inter	Internalizing, sexual health, bullying, school climate	7, 8
Constantine et al. (2015)	Sexuality Education Initiative	Sexual health	Inter	Self-esteem, aggression	9
Cross et al. (2016)	Cyber Friendly School	Cyberbullying	Inter	Bullying	8, 9
Daly et al. (2015)	Yoga	Emotion regulation	Intra	Self-regulation	NR
De Graaf et al. (2016)	Rock and Water	Sexual aggressive behavior and cognitions	Inter	Self-esteem, self-regulation, sexual health	9, 10
De Villiers and Van den Berg (2012)	Resilience program	Resilience	Intra	Resilience, self-esteem, self-regulation, social competence	6
Domino (2013)	Take the lead	Social skills	Inter	Bullying	7
DuRant et al. (1996)	Violence prevention curriculum for adolescents	Violence use	Inter	Aggression	6–8
DuRant et al. (1996)	Conflict resolution: A curriculum for youth providers	Violence use	Inter	Aggression	6–8
Espelage et al. (2013)	Second Step: Student Success Through Prevention	Violence	Inter	Aggression, sexual health, bullying	6
Felver et al. (2018)	Learning to BREATHE	Wellbeing and learning	Intra	Resilience	9–12
Foshee et al. (2005)	Safe Dates	Dating violence	Inter	Aggression	8, 9
Frank et al. (2016)	Transformative life skills	Stress and social-emotional health	Intra	Self-regulation, wellbeing, aggression, school climate	6, 9
Freire et al. (2018)	Challenge: To Be +	Positive development	Intra and inter	Self-esteem, wellbeing	9
Garaigordobil (2004)	Psychological intervention carried out with groups of adolescents	Emotional development	Intra	Self-esteem, self-regulation, internalizing, social competence	NR
Garaigordobil and Martinez-Valderrey (2015a), Garaigordobil and Martinez-Valderrey (2015b) and Garaigordobil (2014)	Cyberprogram 2.0	Interpersonal conflicts and self-esteem	Intra and inter	Self-esteem, internalizing, social competence, bullying, aggression	9, 10
Gardner et al. (2004)	Connections: Relationships and Marriage	Healthy romantic relations	Inter	Aggression, sexual health	11, 12
Ghahremani et al. (2013)	Youth empowerment SEminar (YES)	Emotional wellbeing	Intra	Self-regulation	7–12
Gianotta et al. (2009)	Expressive writing	Negative outcomes associated with peer-related problems	Inter	Self-regulation, bullying	7
Gigantesco et al. (2015)	Definizione di obiettivi e soluzione di problemi (establishing goals and problems solving)	Self-efficacy, psychological wellbeing, and life satisfaction	Intra	Self-regulation, wellbeing	9–11
Gollwitzer et al. (2007)	Vienna Social Competence Training (ViSC)	Class commitment, responsibility, and nonaggressive behavior in conflict	Inter	Aggression	6–8
Gouda et al. (2016)	Mindfulness-based stress education group program	Performance pressure	Intra	Self-esteem, self-regulation, internalizing, wellbeing, social competence	11
Haines and Ellmann (1994)	Stress inoculation training	Negative arousal in response to stress	Intra	Internalizing, wellbeing, aggression	9–12
Hains and Szyjakowski (1990)	Cognitive intervention training program	Cope with stress and negative arousal	Intra	Self-esteem, internalizing, aggression	11, 12
Hains (1994)	Stress inoculation training	Negative arousal in response to stress	Intra	Self-esteem, internalizing, wellbeing, aggression	NR
Haynes and Avery (1979)	Communication skills training program	Self-disclosure and empathy	Intra	Self-esteem, social competence	11
Horn et al. (2010)	JES! Jugendpräventionsprogramm mit Expressivem Schreiben	Emotion regulation	Intra	Wellbeing	8
Huppert and Johnson (2010)	Mindfulness-based stress reduction	Mindfulness, resilience, and psychological wellbeing	Intra	Resilience, wellbeing	8
Ingram et al. (2019)	Stand up: Virtual reality to activate bystanders against bullying	Bullying	Inter	Aggression, social competence, bullying, school climate	7, 8
Jaycox et al. (2006)	Ending violence: A curriculum for educating teens on domestic violence and the las	Intimate partner violence	Inter	Sexual health	9
Jiménez-Barbero et al. (2016)	Count on me	Bullying and violence	Inter	Aggression	7, 8
Kasler et al. (2013)	Meaning of Life program (Israeli adaptation of the Laws of Life program)	Meaning in life	Intra	Self-esteem, wellbeing, school climate	10, 11
Kaveh et al. (2014)	Peer led training program	Self-esteem	Intra	Self-esteem	7
Khanna and Singh (2016)	Nice Thinking (adjusted to Indian culture)	Gratitude and wellbeing	Intra	Wellbeing	7
Kiselica et al. (1994)	Stress inoculation training with assertiveness training	Anxiety and stress	Intra	Internalizing, wellbeing	9
Klingman and Horchdof (1993)	Cognitive-behavioral oriented distress-coping training	Distress-coping	Intra	Wellbeing, social competence	8
Kozina (2018a) and Kozina (2018b)	My FRIENDS	Anxiety	Intra	Internalizing, aggression	8
Lamke et al. (1988)	Cognitive-behavior modification program	Self-statements and self-esteem	Intra	Self-esteem	9
Macgowan (1997)	Dating violence prevention program	Dating violence	Inter	Aggression	6–8
Menesini et al. (2003)	Peer support model	Bullying	Inter	Bullying	6–8
Muck et al. (2018)	Scientist practitioner program	Sexual violence	Inter	Sexual health	8, 9
Nash (2007)	Empower youth program (and Usual school services)	Health, wellbeing, and optimism for futures	Intra	Internalizing	6–8
Noggle et al. (2012)	Kripalu yoga	Overall wellbeing	Intra	Resilience, wellbeing, aggression	11, 12
Orpinas et al. (1995)	Second Step: A violence prevention curriculum	Violence	Inter	Self-esteem, aggression	6
Ortega-Barón et al. (2019)	Prev@cib	Bullying and cyberbullying	Inter	Bullying	7–10
Pacifici et al. (2001)	Dating and Sexual Responsibility	Dating violence	Inter	Sexual health	10
Proctor et al. (2011)	Strengths Gym	Build strengths and learn new strengths	Intra	Wellbeing, self-esteem	7, 8
Richardson et al. (2009)	BodyThink	Self-esteem	Intra	Self-esteem, bullying	7
Ruini et al. (2006)	Cognitive Behavioral Therapy	Mood disorder and psychobiological distress	Intra	Resilience, self-esteem, self-regulation, internalizing, wellbeing, aggression, social competence	NR
Ruini et al. (2006)	Well-being therapy (WBT)	Psychological wellbeing	Intra	Resilience, self-esteem, self-regulation, internalizing, wellbeing, aggression, social competence	NR
Ruini et al. (2009)	Well-being Therapy (WBT) with added cognitive-behavioral packages	Psychological wellbeing and optimal functioning	Intra	Self-regulation, internalizing, wellbeing, aggression, social competence	9, 10
Ruiz-Aranda et al. (2012)	INTEMO	Aggressive behaviors, psychosocial maladjustment, and mental health	Intra and inter	Self-esteem, internalizing, wellbeing, social competence	7
Sánchez-Jiménez et al. (2018)	Dat-e Adolescence	Dating violence	Inter	Self-esteem, aggression, sexual health	7–10
Schramm and Gomez-Scott (2012)	Relationship Smarts Plus	Healthy romantic relationships	Inter	Sexual health	8–12
Schultz et al. (2001)	Facing History and Ourselves	Perspective-taking, critical thinking, and more decisions	Inter	Self-esteem, aggression, social competence	8
Shek and Ma (2011)	Positive Adolescent Training through Holistic Social Programs (P.A.T.H.S.)	Holistic youth development	Intra and inter	Resilience, self-esteem, self-regulation, social competence	7
Shinde et al. (2018)	Strengthening evidence base on school-based interventions for promoting adolescent health (SEHER)	School climate and health-promoting behaviors	Intra and inter	Internalizing, aggression, bullying, school climate	8
Shoshani and Steinmetz (2014)	Maytiv School Program	Mental health and empowerment	Intra	Self-esteem, wellbeing	7–9
Sibinga et al. (2013)	Mindfulness-based stress reduction program	Psychological symptoms and coping	Intra	Internalizing, aggression	7, 8
Simons-Morton et al. (2005)	Going Places Program	Social skills and problem behaviors	Inter	Aggression	6
Soliday et al. (2004)	Expressive writing Intervention	Positive functioning and stress	Intra	Internalizing, wellbeing	8
Solomontos-Kountouri et al. (2016)	ViSC social competence program (with added parental component)	Victimization and aggressive behavior	Inter	Aggression, bullying	7, 8
Sorrentino et al. (2018)	Tabby Improved Prevention and Intervention Program (TIPIP)	Cyberbullying and victimization	Inter	Bullying	NR
Stevens et al. (2000)	Flemish anti-bullying program	Bullying and victimization	Inter	Social competence, bullying	NR
Thomaes et al. (2009)	Self-affirmation intervention	Narcissistic aggression	Inter	Self-esteem, aggression	7, 8
Thompkins et al. (2014)	Violence Prevention Project	Conflict resolution skills	Inter	Social competence	9, 10
Tomyn et al. (2016)	Think Health and Wellbeing	Thinking style, self-esteem, and resilience	Intra	Resilience, self-esteem, internalizing	8
Trip et al. (2015)	Rational Emotive Behavioral Education (REBE)	Negative dysfunctional emotions and alternative to low frustration tolerance	Intra	Aggression, bullying	6
Trip et al. (2015) and Yanagida et al. (2019)	Viennese Social Competence (ViSC)	Bullying and aggressive behavior	Inter	Aggression, bullying	6, 7
Tunariu et al. (2017)	iNEAR	Positive identities, character strengths, and resilience	Intra	Wellbeing, social competence	6, 7
Van der Meulen et al. (2010)	Adjusted version of EQUIP program for Educators	Peer victimization	Inter	Sexual health, bullying, school climate	8, 9
Van Schoiack-Edstrom et al. (2002)	The Second Step, Middle school/Junior High program	Prosocial skills and impulsive-aggressive behavior	Inter	Aggression, social competence	6, 7
Williams et al. (2015) and Miller et al. (2015)	Start strong: Building healthy teen relationships	Healthy romantic relationships and dating violence	Inter	Aggression, bullying, sexual health, social competence	7
Williford et al. (2013)	KiVa antibullying program	Cyberbullying and victimization	Inter	Bullying	8, 9
Wong et al. (2011)	Restorative Whole-school Approach	Bullying	Inter	Self-esteem, social competence, bullying, school climate	7–9
Yom and Eun (2005)	Educational Program for the Prevention of Sexual Violence	Sexual violence	Inter	Sexual health	6

*Intra* intrapersonal domain, *Inter* interpersonal domain, *NR* not reported

## Appendix E—Definitions of the General Domains and the Subdomains

### Intrapersonal Domain

The ability to manage one’s own feelings, emotions, and attitudes about the self (Barber 2005). This domain concerns the subjective processing of behaviors, thoughts, and emotions pertained by the individual self (Dufner et al. 2019; Finkel and Vohs 2006). Evaluating and regulating one’s own inner world and experiences can facilitate positive personal functioning (e.g., psychological wellbeing and resilience), whereas difficulties in this process can increase the chance of developing psychological problems (e.g., internalizing behavior; Dufner et al. 2019).

- *Resilience* the ability to bounce back from the variety of challenges that can arise in life (Campbell-Sills and Stein 2007).
- *Self-esteem* the extent to which individuals like themselves as a person in general or on specific domains (Wichstraum 1995).
- *Self-regulation* the automatic or deliberate modulation of affect, behavior, and cognition (Karoly 1993).
- *General wellbeing* the presence of positive emotions and life satisfaction, and the absence of negative feelings (Robitail et al. 2007).
- *Internalizing problems* a broad range of mood and anxiety behaviors directed towards one’s inner world putting an individual at risk of developing later mood and anxiety disorders (Petty et al. 2008).

### Interpersonal Domain

The ability to build and maintain positive relationships with others, to understand social situations, roles and norms, and to respond appropriately (Pellegrino and Hilton 2012; Shek and Leung 2016). By planning one’s own behavior and predicting the behavior of others one can act in a socially appealing way, such as building positive interpersonal relations, or in a more destructive way, such as behaving aggressively or bullying (Finkel and Vohs 2006).

- *Sexual health* one’s coping skills and attitudes in romantic situations and relations (De Graaf et al. 2005).
- *Social competence* skills to successfully and positively interact with others in social situations (Shek and Leung 2016).
- *School climate* relationships among students and school staff within the school and behavioral norms, goals, and values that engender feelings of safety (Hopson et al. 2014).
- *Aggression* intentional, proactive or reactive, behavior intended to hurt. Others (Coyne et al. 2010).
- *Bullying* repeated, over time, exposure to negative actions (physical, verbal, or nonverbal) with the intention to hurt or bring discomfort upon another by one or more other individuals who are stronger (i.e., imbalance in strength; Olweus 1993).
